# Impact of fluid balance and opioid-sparing anesthesia within enchanced recovery pathway on postoperative morbidity after transthoracic esophagectomy for cancer

**DOI:** 10.3389/fmed.2024.1366438

**Published:** 2024-05-06

**Authors:** Marija Djukanovic, Ognjan Skrobic, Dejan Stojakov, Nebojsa Nick Knezevic, Biljana Milicic, Predrag Sabljak, Aleksandar Simic, Marija Milenkovic, Svetlana Sreckovic, Dejan Markovic, Ivan Palibrk

**Affiliations:** ^1^School of Medicine, University of Belgrade, Belgrade, Serbia; ^2^Department of Anaesthesiology and Intensive Care, Hospital for Digestive Surgery, University Clinical Center of Serbia, Belgrade, Serbia; ^3^Department of Esophagogastric Surgery, Hospital for Digestive Surgery, University Clinical Center of Serbia, Belgrade, Serbia; ^4^Surgery Clinic, Clinical Centre “Dr. Dragisa Misovic – Dedinje”, Belgrade, Serbia; ^5^Department of Anaesthesiology, Advocate Illinois Masonic Medical Center, Chicago, IL, United States; ^6^Department of Anaesthesiology, College of Medicine, University of Illinois, Chicago, IL, United States; ^7^Department of Surgery, College of Medicine, University of Illinois, Chicago, IL, United States; ^8^Department of Medical Statistics and Informatics, School of Dental Medicine, University of Belgrade, Belgrade, Serbia; ^9^Department of Anaesthesiology, Emergency Center, University Clinical Center of Serbia, Belgrade, Serbia; ^10^Department of Anesthesiology, Clinic for Orthopedics Surgery and Traumatology, University Clinical Center of Serbia, Belgrade, Serbia; ^11^Department of Cardiac Anesthesiology, Hospital of Cardiovascular Surgery, University Clinical Center of Serbia, Belgrade, Serbia

**Keywords:** enhanced recovery after surgery, goal-directed therapy, fluid balance, opioid-sparing anesthesia, morbidity, transthoracic esophagectomy

## Abstract

**Background:**

Enhanced Recovery After Surgery (ERAS) protocol for esophagectomy may reduce the high incidence of postoperative morbidity and mortality. The aim of this study was to assess the impact of properly conducted ERAS protocol with specific emphasis on fluid balance and opioid-sparing anesthesia (OSA) on postoperative major morbidity and mortality after esophagectomy.

**Methods:**

Patients undergoing elective esophagectomy for esophageal cancer at the Hospital for Digestive Surgery, University Clinical Center of Serbia, from December 2017 to March 2021, were included in this retrospective observational study. Patients were divided into two groups: the ERAS group (OSA, intraoperative goal-directed therapy, and postoperative “near-zero” fluid balance) and the control group (opioid-based anesthesia, maintenance mean blood pressure ≥ 65 mmHg, and liberal postoperative fluid management). The primary outcome was major morbidity within 30 days from surgery and 30-day and 90-day mortality. Multivariable analysis was used to examine the effect of the ERAS protocol.

**Results:**

A total of 121 patients were divided into the ERAS group (69 patients) and the control group (52 patients). Patients in the ERAS group was received less fentanyl, median 300 (interquartile range (IQR), 200–1,550) mcg than in control group, median 1,100 (IQR, 650–1750) mcg, *p* < 0.001. Median intraoperative total infusion was lower in the ERAS group, 2000 (IQR, 1000–3,750) mL compared to control group, 3,500 (IQR, 2000–5,500) mL, *p* < 0.001. However, intraoperative norepinephrine infusion was more administered in the ERAS group (52.2% vs. 7.7%, *p* < 0.001). On postoperative day 1, median cumulative fluid balance was 2,215 (IQR, −150-5880) mL in the ERAS group vs. 4692.5 (IQR, 1770–10,060) mL in the control group, *p* = 0.002. After the implementation of the ERAS protocol, major morbidity was less frequent in the ERAS group than in the control group (18.8% vs. 75%, *p* < 0.001). There was no statistical significant difference in 30-day and 90-day mortality (*p* = 0.07 and *p* = 0.119, respectively). The probability of postoperative major morbidity and interstitial pulmonary edema were higher in control group (OR 5.637; CI95%:1.178–10.98; *p* = 0.030 and OR 5.955; CI95% 1.702–9.084; *p* < 0.001, respectively).

**Conclusion:**

A major morbidity and interstitial pulmonary edema after esophagectomy were decreased after the implementation of the ERAS protocol, without impact on overall mortality.

## 1 Introduction

Esophageal cancer is the seventh-ranked cancer in the world with an incidence of 572,034 new cases, and the sixth in overall mortality with 508,585 deaths per year (1). Esophagectomy remains the only potentially curative treatment option for patients with cancer invading more than mucosa. However, perioperative morbidity is still high with significant mortality, even in high-volume centers.

Although a multidisciplinary team is required during the perioperative treatment of esophageal cancer, adopted clinical pathways vary significantly between institutions. Changing these well-established and experience-based protocols is difficult. There is a growing interest related to the positive impact of Enhanced Recovery After Surgery (ERAS) protocol for esophagectomy on postoperative morbidity (1–4). However, ERAS is a challenging protocol requiring close work of surgeons and anesthesiologists, along with nurses and physiotherapists. The role of an anesthesiologist during the perioperative period is one of the most important factors in the proper implementation of ERAS protocol for esophagectomy. Specific elements of ERAS protocol such as opioid-sparing anesthesia, intraoperative goal-directed therapy (GDT), and postoperative „near-zero “fluid balance should be implemented by well-trained, experienced, and dedicated anesthesiologist (4–8). A few studies investigate the impact of these elements and the role of the anesthesiologist in the ERAS protocol (3, 9–11). However, the implementations of these elements in the ERAS protocol have been still fully underexplored. We wanted to show the impact of the implementation of ERAS protocol with anesthesiologists’ elements as an important role in everyday practice on clinical outcomes following esophagectomy.

The aim of this study was to assess the impact of properly conducted ERAS protocol with specific emphasis on fluid management (GDT and “near-zero” fluid balance) and opioid-sparing anesthesia on postoperative morbidity and mortality after esophagectomy, and to compare it with the usual clinical pathway.

## 2 Methods

### 2.1 Study population

This is a single-center clinical retrospective observational study. All consecutive patients undergoing elective esophagectomy for esophageal adenocarcinoma (EAC) or squamocellular carcinoma (SCC) at Hospital for Digestive Surgery, University Clinical Center of Serbia, from December 2017 to March 2021, were assessed for eligibility. Patients between 18 and 80 years old with gastric conduit reconstruction after esophagectomy were included. Exclusion criteria were chronic renal failure with permanent hemodialysis, and loss to complete follow-up. The present study was approved by the Ethical Committee of the University Clinical Center Serbia (number 88/46). Written informed consent was obtained from all study participants. This manuscript adheres to the applicable Strengthening the Reporting of Observational Studies in Epidemiology (STROBE) guidelines.

Patients were divided into two groups according to the implementation of the ERAS protocol. Patients with completed ERAS protocol were assigned to the ERAS group, and patients with standard care after esophagectomy were assigned to the control group. Anesthesia and postoperative treatment in the ERAS group were delivered and guided by three anesthesiologists, previously trained in ERAS protocol and using goal-directed therapy monitoring, from January 2017 to November 2017. This period known as the learning and implementation curve was not included in the study. Standard of care anesthesia was performed by all other anesthesiologists, and postoperative treatment was performed by a surgeon in the control group. Patients were assigned to the ERAS group or the control group the day before planned surgery according to the schedule of attending anesthesiologist in the operating room. An anesthesiologist’s schedule for the operating room was made randomly.

A subtotal esophagectomy and gastric conduit reconstruction by intrathoracic (Ivor Lewis) or cervical esophago-gastric anastomosis (McKeown) were performed in all patients. In a few cases lower intrathoracic esophago-gastric anastomosis was created according to Garlock-Sweet, directly influenced by conduit vascularization. The surgical approach was open or minimally invasive. The minimally invasive approach included a hybrid procedure (laparoscopy followed by right thoracotomy) or total minimally invasive esophagectomy – tMIE (laparoscopy followed by thoracoscopy). Two-field standard lymphadenectomy was the standard of care. A median width of gastric conduit was preferable option, and a circular stapler was always used for performing esophago-gastric anastomosis in the upper mediastinum or a linear stapler in the neck. All surgical procedures were performed by experienced surgeons (volume over 20 esophagectomies per year per individual surgeon).

### 2.2 ERAS protocol and standard of care protocol

In our hospital, the ERAS protocol for esophagectomy was introduced in January 2017, but without implementation, all anesthesiologists’ elements (opioid-sparing anesthesia and fluid management) in all patients. Preadmission – evaluation by an anesthesiologist was obligatory for all patients. All preoperative, intraoperative, and postoperative components of ERAS protocol as well as our standard care are listed in Table 1. On admission, the Nutrition Risk Screening 2002 (NRS-2002) score was used for nutritional risk assessment of all patients. If the NRS-2002 score was ≥3, preoperative nutrition support was started. Oral nutrition supplements, enteral and/or parenteral nutrition were initiated depending on esophageal obstruction. Patients from both groups received 1,000 mL of Hartman solution on the day before surgery regardless of the permission to eat. In the ERAS group, thoracic epidural anesthesia was obligatory, except for those with absolute contraindication for epidural catheter placement. A thoracic epidural catheter was inserted before the induction of general anesthesia (Table 1). A test dose of 2 mL lidocaine 2% was administered for confirmation of accurate placement. In both groups of patients, general anesthesia was induced with propofol, fentanyl, and rocuronium, and maintained with sevoflurane. Depth of anesthesia was adjusted to bispectral index (BIS) to 40–60 in both groups. In ERAS group, after induction, a bolus of 5-10 mL levobupivacaine 0.25% was administered and intraoperative pain control was obtained with continuous epidural analgesia (levobupivacaine 0.125% + fentanyl 2mcg/mL), 5–12 mL/h, based on the patient’s weight and level of comfort. In control group or in case of absence of epidural catheter in patients in the ERAS group, intraoperative pain control was achieved with intravenous fentanyl boluses, based on the patient’s weight and needs for additional analgesia. Patients were ventilated with a tidal volume (Vt) of 7–8 mL/kg (ideal body weight) and PEEP≥5cmH_2_O. During the transthoracic procedure, one-lung ventilation (OLV) was applied whenever was tolerated by a patient. In the course of OLV, protective mechanical ventilation was provided (Table 1). The frequency of ventilation was controlled to the end-tidal carbon dioxide value of 3.5-6 kPa. An arterial line was inserted in all patients. Baseline Hartmann’s solution was administered at a rate of 3 mL/kg/h in the first hour in both groups with an intraoperative maintains fluid rate of 1 mL/kg/h. In the ERAS group, goal-directed therapy was provided by LiDCORapid™ (LiDCO Ltd., Cambridge, UK) after connection with the arterial line. Hemodynamic parameters – invasive arterial blood pressure (IABP), cardiac output (CO), cardiac index (CI), stroke volume (SV), stroke volume index (SVI), stroke volume variation (SVV), systemic vascular resistance (SVR) and systemic vascular resistance index (SVRI) were measured using LiDCORapid™. The goal was mean arterial pressure (MAP) ≥ 65 mmHg, SVV < 13% in the absence of arrhythmia or maintance SVI within 10% of baseline, respecting fluid tolerance. The fluid challenge with crystalloids (Hartmann’s solution 250 mL) was administered only MAP ≤65 mmHg and SVI decreased by 10% and more from baseline and SVV rose, without trying to achieve full fluid responsiveness. The administration algorithm for an albumin solution (5% 250 mL) was SVV > 13% in the abdominal phase of surgery or SVI < 33 mL/m^2^ in the thoracic phase of surgery. Fluid challenge was continued with crystalloids if SVV was still >13% after a bolus of albumin 5% or SVI was still <33 mL/m^2^. If MAP ≤65 mmHg, with stable baseline SVI and SVV < 10%, norepinephrine was administered. Before administration of norepinephrine, pneumothorax had to be excluded. In the control group, the goal of intraoperative hemodynamic management was maintained at MAP ≥65 mmHg while fluid administration or norepinephrine was dependent upon the attending anesthesiologist. At the end of the surgery, the patient was extubated whenever possible in both groups. In the ERAS group, postoperative care had to fulfill all elements from Table 1. In the control group, the standard of care was current practice in our hospital (Table 1). In the ERAS group, the postoperative goal was to keep the “near-zero” fluid balance, respecting MAP ≥65 mmHg, capillary refill <2 s, central venous oxygen saturation (ScvO_2_) ≥ 65%, and urine output ≥0.5 mL/kg/h. If needed, the vasopressor of choice was norepinephrine. In the control group, postoperative fluid therapy had been administered according to the individual judgment by the intensivist in charge in the ICU or attending surgeon in a ward, to achieve MAP ≥65 mmHg and urine output ≥0.5 mL/kg/h.

**Table 1:** Treatment protocol in ERAS and control group.

Intervention	ERAS group	CONTROL group
**Preoperative period**		
Preadmission counseling	Obligatory	
Nutritional risk assessment	Nutrition Risk Screening 2002 (NRS 2002)	
Preoperative nutrition therapy	Patients with NRS 2002 ≥ 3, started with preoperative nutritional support (sip, enteral or parenteral nutrition).	
Cardiopulmonary assessment	6 min test walk (6MTW); heart ultrasound, if needed	
Respiratory physical therapy	Patients with chronic obstructive pulmonary disease (COPD)	
Avoidance of preoperative fasting	Solid food and clear fluid (preoperative high – high-carbohydrate drinks) intake were allowed until 12 and 2 h before surgery, respectively, except for patients with esophageal obstruction	
	All	Seldom
No routine mechanical bowel preparation	Obligatory	
Timing of surgery following neoadjuvant therapy	The time to wait for surgery was a minimum of 3–6 weeks	
**Operative period**		
Prophylactic antibiotic	30 min before surgery in all patients (second-generation cephalosporin)	
Minimally invasive or open	Tubulized stomach, with two-field lymphadenectomy	
Avoidance of benzodiazepines	No benzodiazepines administration	Premedication with benzodiazepines
Anesthetic management	Volatile anesthetic, bispectral index (BIS)	
Opioid-sparing anesthesia	Thoracic epidural analgesia, the level between T5 and T8	No opioid-sparing anesthesia (Opioid analgesia)
Goal-directed therapy	Goal-directed therapy (LiDCO rapid)	Without goal-directed therapy
One-lung ventilation (OLV)	Vt-5-6 mL/kgPBW, Ppeak-35cmH_2_O, Pdriving≤15cmH_2_O, PEEP>5cmH_2_O ventilated lung, Recruitment maneuver after OLV	
	All	Seldom
Intraoperative warming	Maintenance body temperature ≥ 36C°	
Extubation in the operating room	Extubation at the end of surgery	
	All	Seldom
**Postoperative period**		
Early mobilization	On 1 POD	From POD 2
Avoidance of fluids overloads	Maintenance postoperative “near-zero” fluid balance	Liberally fluids therapy
Early removal of nasogastric decompression	On POD 2	
	All	Seldom
Early removal of chest tube	On POD from 3 to 5	
Postoperative pain control	Thoracic epidural analgesia until POD 3, paracetamol, goal NRS < 3, rescue therapy: metamizole, tramadol, morphine	Morphine, tramadol, paracetamol, metamizole, NSAID, goal NRS < 3
Postoperative early oral nutrition	Oral liquid started from POD2, without standard jejunostomy placement	
	All	Seldom
Postoperative glycemic control	Targeted values ≤10 mmoL/L	
Antithrombotic prophylaxis	LMWH in two divided doses. Treatment was started 12 h before surgery. On POD3, LMWH was not given 12 h before and 4 h after the removal thoracic epidural catheter. LMWH administration was continued until discharge from the hospital	LMWH in two divided doses. Treatment was started 2 h before surgery and continued until discharge from the hospital
Planned ICU discharge	On POD 1	From POD 2

Vt, tidal volume; PBW, predicted body weight; Ppeak, peak inspiratory airway pressure pressure; Pdriving, driving pressure; PEEP, positive end-expiratory pressure; POD, postoperative day; NRS, numerical rating scale; NSAID, non-steroid anti-inflammatory drugs; LMWH, low-molecular-weight heparin.

Postoperative fluid balance was carefully calculated taking into account fluids administered and eliminated through all routes, including blood loss and drainage. Daily fluid balance was calculated by subtracting the fluid eliminated from the total fluid administered from 05 h a.m. to 05 h a.m. the next day. The cumulative fluid balance at postoperative day (POD) 1 was calculated from induction of anesthesia to postoperative day 1 at 05 h, and the cumulative fluid balance at POD 2 was calculated from induction of anesthesia to POD 2 at 05 h.

### 2.3 Outcomes

The primary outcomes were a major morbidity within 30 days from surgery and 30-day and 90-day mortality.

Secondary outcomes were hospital and ICU length of stay (LOS), the incidence of interstitial pulmonary edema, major and minor postoperative pulmonary complications.

### 2.4 Definition of postoperative complications

The frequency of major morbidity, interstitial pulmonary edema, postoperative pneumonia, anastomotic leak, and other complications were followed during the first 30 days after surgery. 30-day and 90-day mortality was defined as any death relating to treatment within 30 and 90 days after surgery, respectively. Interstitial pulmonary edema was defined by chest x-ray and hypoxemia (peripheral blood saturation, SpO_2_ < 91%) in the absence of heart failure. Pneumonia was defined by current guidelines (12). All chest-x rays were examined and radiological diagnoses were made by the radiologist. Major postoperative pulmonary complications (PPC) were defined as pneumonia, acute respiratory distress syndrome, atelectasis, and pleural effusion, empyema or pneumothorax requiring intervention. A minor PPC were defined as atelectasis, pleural effusion or pneumothorax without requiring intervention. Surgical complications were listed as follows: anastomotic leak, gastric necrosis, bleeding, chylothorax, chylous effusion, or any need for surgery for reoperation (12). Major morbidity was presented by a number of patients with developed one or more major complications (major PPC, surgery complications, re-intubation, acute myocardial infarction, pulmonary embolism, renal failure requiring dialysis, sepsis, delirium, new-onset stroke, postoperative arrhythmia, and infection leading to prolonged hospital stay).

### 2.5 Statistics analysis

Descriptive statistics were calculated for demographic characteristics, comorbidity, and other parameters (preoperatively and postoperatively) and were presented as frequencies and proportions. Numeric data were tested for normal distribution using the Kolmogorov–Smirnov test. The Mann–Whitney test was used to compare the nonparametric numeric data. Independent t-test was used to compare parametric numeric data. Categorical data were analyzed using the Pearson chi-square test. Univariable and multivariable logistic regression methods were used for statistical analysis of differences between groups with and without ERAS protocol. All test variables with a statistically significant *p* < 0.05 in the univariable model were included in the multivariable model. Statistical significance was considered at *p* < 0.05. Statistical analysis was performed using the IBP SPSS Statistics v28 (Statistical Package for Social Sciences, SPSS Inc., Chicago, Illinois).

## 3 Results

During the study period, 157 patients who underwent esophagectomy for esophageal carcinoma were assessed for the current study. After excluding three patients older than 80 years, three patients with esophagectomy with colon conduit reconstruction, one patient with esophagectomy with gastric conduit reconstruction and simultaneous colon resection, and 8 patients with palliative surgery, 142 patients were included. According to predefined exclusion criteria, two patients with chronic renal failure on permanent hemodialysis and 19 patients lost from follow-up were excluded from further analysis. The remaining 121 patients were included in the final analysis and divided into two groups: the ERAS group (69 patients) and the control group (52 patients).

The distribution of the ASA (American Society of Anesthesiologists) score was different between groups – patients in the ERAS group were rated with a higher score (*p* = 0.005). BMI (Body Mass Index) was higher in the ERAS group (*p* = 0.019). The distribution of type of surgery (tMIE), Hybrid technics or open surgery was significantly different between groups (*p* = 0.046). Preoperative characteristics and the type of surgery are shown in detail in Table 2. The compliance rate to preoperative components of the ERAS protocol was 90.91%.

**Table 2:** Patients characteristics.

Characteristics	Total number patients *n* = 121	ERAS group *n* = 69	Control group *n* = 52	*p-*value
Sex, male (%)	102 (84.3)	58 (84.1)	44 (84.6)	1.000
Age	65 [29–79]	64 [29–79]	65 [38–78]	0.604
Weight, kg	72 [40–117]	76 [40–117]	70.5 [41–108]	0.100
BMI	23.2 [15.2–39.6]	25.5 [15.2–39.6]	22.1 [16.2–34.6]	0.019
NRS 2002	4 [2–6]	4 [2–6]	4 [2–6]	0.518
ASA status, *n* (%)				0.005
1	2 (1.7)	2 (2.9)	0 (0)	
2	55 (45.5)	23 (33.3)	32 (61.5)	
3	58 (47.9)	38 (55.1)	20 (38.5)	
4	6 (5)	6 (8.7)	0 (0)	
POSSUM	17 [13–38]	18 [13–38]	16 [13–30]	0.315
RCRI	1 [1–3]	1 [1–3]	1 [1–3]	0.535
Pack-year	23 [0–120]	23 [0–120]	25 [0–90]	0.524
Hypertension, *n* (%)	61 (50.4)	35 (50.7)	26 (50)	1.000
Coronary disease, *n* (%)	11 (9.1)	6 (8.7)	5 (9.6)	1.000
Arrhythmia, *n* (%)	26 (21.5)	14 (20.3)	12 (23.1)	0.824
Diabetes mellitus, *n* (%)	12 (9.9)	5 (7.3)	7 (13.5)	0.358
COPD, *n* (%)	14 (11.6)	9 (13)	5 (9.6)	0.775
Emphysema, *n* (%)	26 (21.5)	13 (18.8)	13 (25)	0.504
Asthma, *n* (%)	5 (4.1)	4 (5.8)	1 (1.9)	0.39
Cerebral vascular disease, *n* (%)	6 (5)	4 (5.8)	2 (3.8)	0.699
Hypothyroidism, *n* (%)	2 (1.7)	2 (3)	0 (0)	0.506
CKD, *n* (%)	6 (5)	5 (7.2)	1 (1.9)	0.235
6MWT, *m*	438 [238–735]	446 [238–735]	426 [305–720]	0.945
TNM stage, *n* (%)				
1/2	30 (24.8)	19 (27.5)	12 (23.1)	0.676
3/4	91 (75.2)	50 (72.5)	40 (76.9)
Neoadjuvant therapy, *n* (%)	26 (21.5)	15 (21.7)	11 (21.2)	0.999
Type of surgery, *n* (%)				
tMIE	57 (47.1)	39 (56.5)	18 (34.6)	0.046
Hibrid IL	40 (33.1)	22 (31.9)	18 (34.6)	
Open Ivor Lewis	17 (14.1)	5 (7.2)	12 (23.1)	
Garloc-Sweet	3 (2.5)	1 (1.4)	2 (3.8)	
Oesophagectomy (McKeown)	4 (3.3)	2 (2.9)	2 (3.8)	

Data are expressed as median [inter-quartile range] unless indicated otherwise. BMI, Body mass index; NRS-2002, Nutrition Risk Screening 2002; ASA, American Society of Anesthesiologists; POSSUM, Physiological and Operative Severity score for the enUmeration of Mortality and Morbidity; RCRI, Revised Cardiac Risk Index; COPD, chronic obstructive pulmonary disease; CKD, chronic kidney disease; 6MWT, 6 Minute Walk Test; TNM, tumor node metastasis; tMIE, totally minimally invasive esophagectomy.

Total operative time was shorter in the ERAS group, median 320 (interquartile range, 185–440) minutes, than in the control group, median 345 (interquartile range, 260–275) minutes, *p* = 0.006. The median operative time of the abdominal phase was shorter in the ERAS group compared to the control group: 155 (interquartile range, 60–240) minutes vs. 197.5 (interquartile range, 80–320) minutes, *p* < 0.001. Thoracic epidural analgesia (TEA) was provided in 75.4% of patients in the ERAS group and no one from the control group received TEA. Intraoperative fentanyl administration was more common in the control, median 1,100 (interquartile range, 650–1750) mcg than in ERAS group, median 300 (interquartile range, 200–1,550) mcg, *p* < 0.001. One-lung ventilation was applied in 91.3% of patients in the ERAS group and in 61.5% in the control group (*p* < 0.001). Patients with fluid therapy guided by LIDCORapid (ERAS group) received lower fluids: median 2000 (interquartile range, 1,000–3,750) mL vs. median 3,500 (interquartile range, 2000–5,500) mL, *p* < 0.001, but intraoperative norepinephrine infusion was more administered in the patients in the ERAS group (52.2% vs. 7.7%, *p* < 0.001). The percentage of patients who were extubated immediately after surgical procedure was statistically higher in the ERAS group (97.1% vs. 23.1%, *p* < 0.001). Overall intraoperative data are presented in Table 3.

**Table 3:** Intraoperative data.

Intraoperative data	Total number patients (121)	ERAS group (69)	Control group (52)	*p-*value
Total operative time, min	340 [185–725]	320 [185–440]	345 [260–725]	0.006
Thoracic operative time, min	150 [80–415]	155 [80–240]	150 [85–415]	0.957
Abdominal operative time, min	160 [65–320]	155 [65–240]	197.5 [80–320]	<0.001
Epidural analgesia, *n* (%)	52 (43)	52 (75.4)	0 (0)	<0.001
Fentanyl, mcg	900 [200–1750]	300 [200–1,550]	1,100 [650–1750]	<0.001
One-lung ventilation, *n* (%)	95 (78.5)	63 (91.3)	32 (61.5)	<0.001
One-lung ventilation time, min	130 [65–275]	130 [65–205]	125 [75–275]	0.712
Total infusion, mL	2,500 [1000–5,500]	2000 [1000–3,750]	3,500 [2000–5,500]	0.001
Total infusion, mL/kg/h	6 [0.6–16.7]	4.9 [0.6–11.6]	8.4 [3.7–16.7]	<0.001
Crystalloid infusion, mL	2,150 [1000–5,000]	1800 [1000–3,000]	3,000 [2000–5,000]	<0.001
Colloid infusion, mL	250 [0–1,000]	250 [0–750]	300 [0–1,000]	<0.001
Norepinephrine administration-bolus of 10mcg, *n* (%)	21 (17.4)	17 (24.6)	4 (7.8)	0.016
Norepinephrine administration continuous, *n* (%)	40 (33.1)	36 (52.2)	4 (7.7)	<0.001
Transfusion, *n* (%)	6 (5)	3 (4.3)	3 (5.8)	1.000
Transfusion amount, mL	257.5 [235–275]	250 [250–265]	265 [235–275]	0.712
Urine output, mL	500 [200–2,350]	500 [200–2000]	500 [200–2,350]	0.483
Extubation at the end of surgery, *n* (%)	79 (65.3)	67 (97.1)	12 (23.1)	<0.001
Hypotension, *n* (%)	17 (14)	10 (14.5)	7 (13.5)	1.000
Hypertension, *n* (%)	24 (19.8)	8 (11.6)	16 (30.8)	0.01
Atrial fibrillation, *n* (%)	2 (1.7)	0 (0)	2 (3.8)	0.183
Acidosis, *n* (%)	41 (33.9)	21 (30.4)	20 (38.5)	0.438
Open contralateral pleura requiring chest tube, *n* (%)	42 (34.7)	27 (39.1)	15 (28.8)	0.255
Surgical complication, *n* (%)	2 (5)	4 (5.8)	2 (3.8)	0.699

Data are expressed as median [inter-quartile range] unless indicated otherwise.

**Figure 1:**
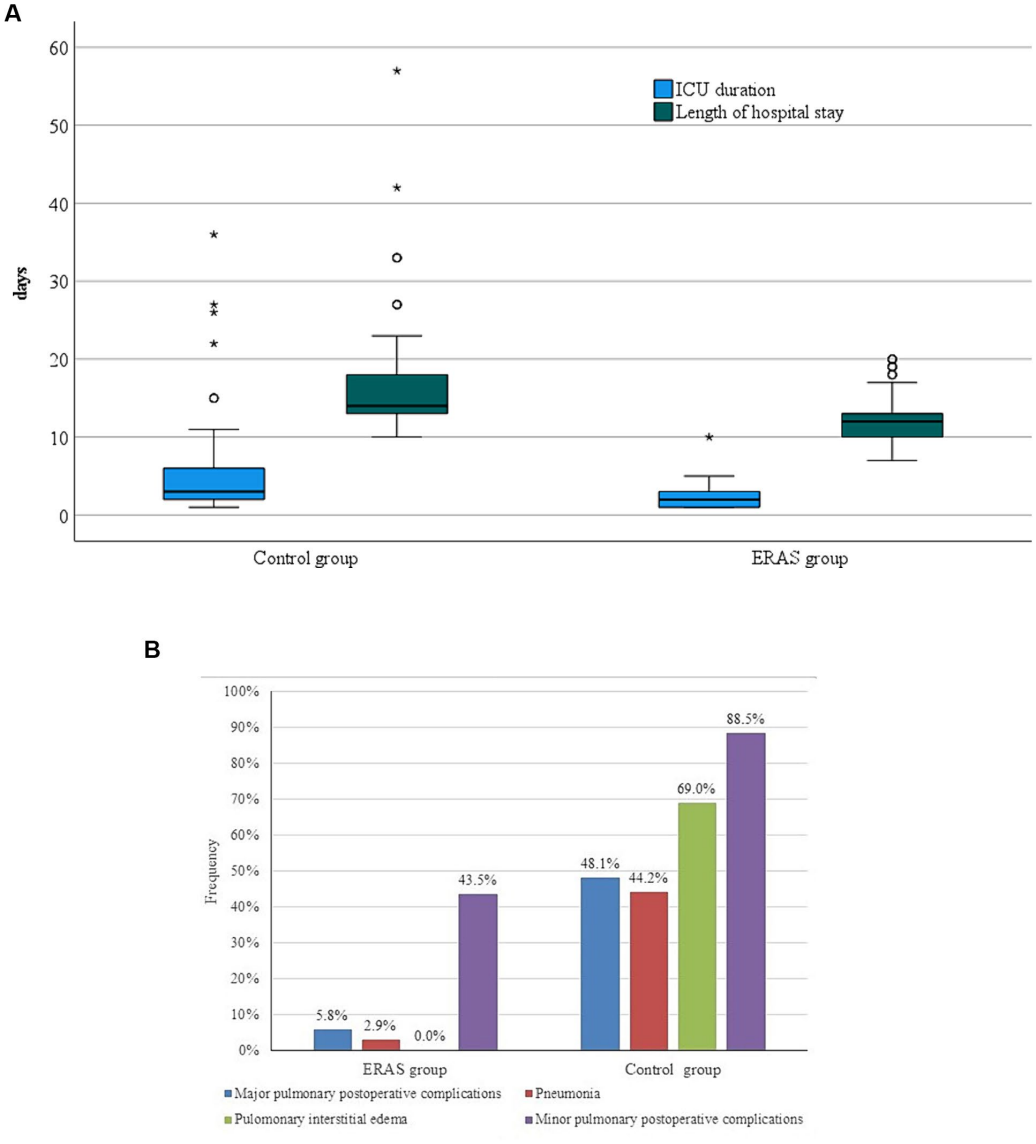
**(A)** ICU duration and hospital stay between groups. **(B)** Postoperative pulmonary complications.

In the postoperative period, median cumulative fluid balance on POD1 was 2,215 (interquartile range, −150–5880) mL in the ERAS group vs. 4692.5 (interquartile range, 1770–10,060) mL in the control group, *p* = 0.002. There was a statistically significant difference in cumulative fluid balance on POD2 between groups, median 2,250 (interquartile range, −1,580–6030) mL in ERAS vs. median 4,990 (1400–9,825) mL in the control group (*p* < 0.001). None of patients from the ERAS group received norepinephrine postoperatively, while 2 patients received norepinephrine postoperatively in the control group. On POD1, 89.9% of patients in the ERAS group were mobilized in contrast 3.8% of patients in the control group (*p* < 0.001) (Table 4). Postoperative morbidity at day 30 were lower in the ERAS group than in the control group (18.8% vs. 75%, *p* < 0.001) (Table 5). There was no significant difference in 30-day and 90-day mortality between groups (*p* = 0.07 and *p* = 0.119, respectively). The length of hospital stay was significantly shorter in the ERAS group, median 12 (interquartile range, 7–20) days than in the control group, median 14 (interquartile range, 10–57) days, *p* < 0.001 (Figure 1A). Occurrence of interstitial pulmonary edema was significantly lower in the ERAS group (0%) compared to the control group (69%), *p* < 0.001. Pneumonia, major postoperative pulmonary complications (Figure 1B) and atrial fibrillation were lower in the ERAS group compared to the control group (2.9% vs. 44.2%, *p* < 0.001; 5.8% vs. 48.1%, *p* < 0.001; 5.8% vs. 19.2%, *p* = 0.041, respectively) (Table 5). Surgical complications were significantly less frequent in the ERAS group (2.9%) than in the control group (21.2%), *p* = 0.002. Anastomotic leakage was confirmed in 7 (13.5%) patients in the control group, while two of them were treated by surgery. There was no anastomotic leakage in the ERAS group. Postoperative bleeding requiring re-operation was registered in one patient in the control group (Table 5). Inguinal hernia incarceration treated by re-operation occurred in one patient from the ERAS group.

**Table 4:** Postoperative outcomes.

All events	Total number patients (121)	ERAS group (69)	Control group (52)	*p-*value
Cumulative fluids balance on POD 1, mL	3,420 [−150–10,060]	2,215 [−150–5,880]	4692.5 [1770–10,060]	0.002
Cumulative fluids balance on POD 2, mL	3,240 [−1,580–9,825]	2,250 [−1,580–6,030]	4,990 [1400–9,825]	<0.001
Norepinephrine on the day of surgery and POD 1–3, *n* (%)	2 (1.7)	0 (0)	2 (3.8)	0.506
Ventilation time after surgery, min	0 [0–840]	0 [0–180]	105 [0–840]	<0.001
Mobilization on POD1	66 (54.5)	62 (89.9)	4 (3.8)	<0.001
Transfusion, *n* (%)	29 (24)	13 (18.8)	16 (30.8)	0.134
Transfusion amount, mL	0 (0–825)	0 (0–538)	0 (0–825)	0.144
Hyperglycemia on admission in ICU, *n* (%)	29 (24)	4 (5.8)	25 (48.1)	<0.001
Acidosis on admission in ICU, *n* (%)	12 (9.9)	1 (1.4)	11 (21.2)	<0.001

Data are expressed as median [inter-quartile range] unless indicated otherwise. POD, postoperative day; ICU, intensive care unit.

**Table 5:** Postoperative complications, mortality, ICU, and hospital stay.

Postoperative events	Total number patients (121)	ERAS group (69)	Control group (52)	*p-*value
ICU duration, days	3 [1–36]	2 [1–10]	3 [1–36]	0.001
Hospital LOS, days	13 [7–57]	12 [7–20]	14 [10–57]	<0.001
Mortality on day 30, *n* (%)	3 (2.5)	0 (0)	3 (5.8)	0.077
Mortality on day 90, *n* (%)	4 (3.3)	0 (0)	4 (7.7)	0.119
Postoperative morbidity at day 30, *n* (%)	52 (43)	13 (18.8)	39 (75)	<0.001
Major PPC, *n* (%)*	29 (24)	4 (5.8)	25 (48.1)	<0.001
Pneumonia, *n* (%)	25 (20.7)	2 (2.9)	23 (44.2)	<0.001
ARDS, *n* (%)	0 (0)	0 (0)	1 (1.9)	0.43
Empyema pleurae, *n* (%)	1 (0.8)	1 (1.4)	0 (0)	0.999
Atelectasis requiring bronchoscopy, *n* (%)	3 (2.5)	1 (1.4)	2 (3.8)	0.576
Pleural effusion requiring drainage, *n* (%)	2 (1.6)	1 (1.4)	1 (1.9)	0.999
Pneumothorax requiring intervention, (%)	5 (4.13)	2 (2.9)	3 (5.8)	0.651
Re-intubation, *n* (%)	4 (3.3)	0 (0)	4 (7.7)	0.032
Interstitial pulmonary edema, *n* (%)	36 (29.8)	0 (0)	36 (69)	<0.001
Atrial fibrillation, *n* (%)	14 (11.6)	4 (5.8)	10 (19.2)	0.041
Other arrhythmia, *n* (%)	4 (3.3)	2 (2.9)	2 (3.8)	0.999
Acute myocardial infarction, *n* (%)	1 (0.8)	0 (0)	1 (1.9)	0.43
Surgical complication, *n* (%)	13 (10.7)	2 (2.9)	11 (21.2)	0.002
Chylothorax, *n* (%)	1 (0.8)	0 (0)	1 (1.9)	0.43
Bleeding, *n* (%)	4 (3.3)	1 (1.4)	3 (5.8)	0.313
Anastomotic leakage, *n* (%)	7 (5.8)	0 (0)	7 (13.5)	0.002
Anastomotic leakage (conservative treatment), *n* (%)	5 (4.1)	0 (0)	5 (9.6)	0.013
Anastomotic leakage requiring surgery, *n* (%)	2 (1.6)	0 (0)	2 (3.8)	0.183
Gastric necrosis, *n* (%)	0 (0)	0 (0)	0 (0)	0.999
Re-operation, *n* (%)	4 (3.3)	1 (1.4)	3 (5.8)	0.313
Sepsis, *n* (%)	3 (2.5)	0 (0)	3 (5.8)	0.077
Pulmonary embolism, *n* (%)	0 (0)	0 (0)	0 (0)	0.999
Renal failure requiring dialysis, *n* (%)	1 (0.8)	0 (0)	1 (1.9)	0.43
New-onset stroke, *n* (%)	0 (0)	0 (0)	0 (0)	0.999
Delirium, *n* (%)	7 (5.8)	1 (1.4)	6 (11.5)	0.041
Infection, *n* (%)	30 (24.8)	13 (18.8)	17 (32.7)	0.092
Minor PPC, *n* (%)*	76 (62.8)	30 (43.5)	46 (88.5)	<0.001
Pneumothorax, *n* (%)	16 (13.2)	8 (11.6)	8 (15.4)	0.595
Pleural effusion, *n* (%)	65 (53.7)	21 (30.8)	44 (84.6)	<0.001
Atelectasis, *n* (%)	26 (21.5)	8 (11.6)	18 (34.6)	0.002

Data are expressed as median [inter-quartile range] unless indicated otherwise. POD, postoperative day; ICU, intensive care unit; hospital LOS, hospital length of stay; PPC, pulmonary postoperative complications; ARDS, acute respiratory distress syndrome. *PPC, number of patients with one or more postoperative pulmonary complications.

In the univariable analysis, patients from the control group were associated with a higher risk of 30-day morbidity (OR 2.923; CI95% 1.41–6.867; *p* < 0.001), prolonged length of hospital stay (OR 1.271; CI95% 1.114–1.45; *p* < 0.001) and interstitial pulmonary edema (OR 3; CI95% 1.495–10.768; *p* < 0.001). In the multivariable analysis, patients from the control group had about six times higher probability for 30-day morbidity (OR 5.637; CI95% 1.178–10.98; *p* = 0.03) and six times higher risk for interstitial pulmonary edema (OR 5.955; CI95% 1.702–9.084; *p* < 0.001) (Table 6).

**Table 6:** Univariable and multivariable analysis of complications after esophagectomy.

Variables	Univariate analysis			Multivariate analysis		
	OR	95%CI Lower-Upper	*p-*values	OR	95% CI Lower-Upper	*p-*values
Morbidity at day 30	2.923	1.41–6.867	<0.001	5.637	1.178–10.98	0.03
Length of hospital stay	1.271	1.114–1.45	<0.001	1.033	0.896–1.19	0.655
Interstitial pulmonary edema on X-ray	3	1.495–10.768	<0.001	5.955	1.702–9.084	<0.001

## 4 Discussion

Since the ERAS protocol for esophagectomy was introduced, many studies have proven its positive effect on postoperative outcomes. However, compliance with all the elements of this protocol for esophagectomy is difficult due to the complexity of surgery and postoperative care, and already established protocols in high-volume centers. In our study, the implementation of ERAS protocol following esophagectomy for cancer led to reduced 30-day postoperative morbidity, interstitial pulmonary edema, pneumonia, anastomotic leakage, ICU duration, and the hospital LOS in univariable analysis. However, multivariable analysis revealed a higher probability of major morbidity and interstitial pulmonary edema in patients from the control group. Tang and colleagues showed favorable short-term outcomes (hospital LOS, postoperative complications, hospitalization cost) following esophagectomy after implementation of ERAS protocol (3). However, the study comparing ERAS protocol with a modified analgesic regimen combined with perioperative GDT, and standard ERAS protocol did not affect the hospital LoS and the incidence of postoperative complications such as pneumonia and anastomotic leak (9).

The effects of intraoperative GDT are in the focus of research during the past few years. Excess fluid administration could lead to interstitial water retention and lung edema (13). In the randomized control trial by Mukai et al., the implementation of intraoperative GDT reduced overall morbidity and mortality, and shortened hospital stay, without effect on pneumonia and anastomotic leakage in patients after transthoracic esophagectomy (10). A lower incidence of pneumonia, shorter ICU stay were registered after GDT during esophagectomy in the study by Veelo et al., while length of hospital stay, overall morbidity and mortality were similar (14). In our study, ERAS protocol with intraoperative GDT and postoperative “near-zero” fluid balance reduced 30-day major morbidity and interstitial pulmonary edema, with no effect on the incidence of postoperative pneumonia. However, some studies and meta-analyses failed to show a positive impact of intraoperative GDT on the incidence of postoperative complications after surgery of the foregut (15–17). Total intraoperative infusion was significantly less in the ERAS group with intraoperative GDT. The present study showed that patients with TEA and GDT received less fluids despite epidural analgesia but more vasopressors intraoperatively than patients in the control group. Ischemic impact of vasopressors on a gastric conduit was previously described, with a strong recommendation against the application of these drugs during esophagectomy (18). Nevertheless, in more recent study, an intraoperative application of vasopressors was not associated with increased incidence of an anastomotic leak following open Ivor Lewis esophagectomy (19). Similarly, in our study, none of the patients in the ERAS group with intraoperative norepinephrine infusion developed an anastomotic leakage. At the end of the surgical procedure, norepinephrine was suspended in all patients in the ERAS group. Reasons for a good outcome may be found in intraoperative fluids optimization before starting intraoperative norepinephrine infusion due to hypotension, as a consequence of vasodilatation which is an adverse effect of TEA.

High positive cumulative postoperative balance on POD1 was found to be an independent risk factor for postoperative pulmonary complications in high-volume centers (11, 20). In the present study, the cumulative fluid balance on POD1 was significantly lower in the ERAS group than in the control group. In the study from Japan, positive fluid balance higher than 3,000 mL at POD 1 had a significantly negative impact on anastomotic leakage and postoperative pneumonia (21). Besides intraoperative GDT, this might be another explanation of why patients in the ERAS group experienced less postoperative interstitial pulmonary edema and major morbidity. Therefore, optimal perioperative fluid management including an intraoperative GDT and cumulative “near-zero” fluid balance during esophagectomy is one of the most important elements of ERAS protocol.

The fluid shift from intravascular to interstitial space due to endothelial glycocalyx damage is common during perioperative period, in particular in major surgery. The surgical stress causes release of inflammatory mediators (tumor necrosis factor α, interleukins, proteases). Moreover, during iatrogenic acute hypervolemia, atrial natriuretic peptide is released and may degrade endothelial glycocalyx further, increased vascular permeability and made fluid and protein shift from intravascular space toward the interstitium. To protect the endothelial glycocalyx, fluid should be administered carefully and only when relative hypovolemia arises. Intraoperative goal directed therapy may help anesthesiologist to administer fluid only when needed and start with vasopressors (norepinephrine) application early, but safely (22, 23). One-lung ventilation (ischemia–reperfusion injury) and two-field lymphadenectomy in combination with fluid overload could increase the risk for interstitial pulmonary edema and lung injury (24). In early postoperative period (48–72 h), neuroendocrine and metabolic effects on surgical stress are the most pronounced. Following esophagectomy, inflammatory cytokines are released which leads to vasodilatation and increased permeability of the endothelium, and resulting tissue edema, in particular lung edema. Protocol with postoperative “near-zero” fluid balance allow maintaining of euvolemia in the early postoperative period and may decrease postoperative interstitial pulmonary edema, and pneumonia. Postoperative continuous thoracic epidural analgesia up to 72 h may reduce the neuroendocrine stress response after surgery and together with optimal fluid management reduce incidence of postoperative complications (22, 23).

Interstitial pulmonary edema was presented as separately complication. We did not count interstitial pulmonary edema in major PPC, despite it should be according some guidelines, because we believe interstitial pulmonary edema is very important clinical finding, and warning sign we need to adjust current fluid management to prevent further complications (positive fluid balance has to change to zero or negative fluid balance).

In the present study, in ERAS group the postoperative pneumonia was diagnosed only in 2.9% patients with no interstitial pulmonary edema, which is very low incidence. In control group, incidence of pneumonia was 44.2 and 69% interstitial pulmonary edema. Casado et al. have been demonstrated that fluid excess during the perioperative period is a predictor of the development of respiratory complications following esophagectomy (13). However, according current guidelines, the diagnosis of pneumonia is complicated because it involves several pulmonary radiological features, hypoxemia and a high leucocyte count which may be related to postoperative inflammatory response to the surgery, not infection (25). It appears that interstitial pulmonary edema and hypoxemia in combination with postoperative leukocytosis could lead to a false positive diagnosis for pneumonia. This could be one of reason for high incidence of pneumonia in control group.

The next important step in ERAS protocol is adequate perioperative pain management with opioid-sparing anesthesia (3, 4, 26). Opioid-sparing anesthesia is supported in enhanced pathways to avoid adverse effects of opioids such as postoperative nausea and vomiting, intestinal paresis, prolonged wakening from anesthesia, and increased cancer recurrence (27). In the present study, the amount of intraoperatively administered fentanyl was significantly reduced by using thoracic epidural analgesia. TEA with tracheal extubation at the end of the surgical procedure has been proven as a safe procedure, associated with low morbidity and mortality (28). The immediate postoperative extubation rate was 97.1% in the ERAS group, with an 89.9% rate of mobilization on POD1. Postoperative “near-zero” fluid balance and thoracic epidural analgesia are very important for the early mobilization of patients on POD1 (11, 20, 28, 29). Repeatedly, studies showed that immediate extubation in patients with TEA and early mobilization may reduce the systemic inflammatory response and pulmonary complications (28, 30–32).

Implementation of the ERAS protocol in esophageal surgery is very difficult due to the complexity of esophagectomy and patients’ comorbidities. In the present study, the most difficult barrer was persuading the healthcare providers to abandon the previous protocol, start to educate, and switch to a new one, particularly in patients with a higher ASA score. During changing the perioperative routine, checklist is needed to conduct the ERAS protocol with high adherence. Everyday discussion about clinical findings need to be done between anesthesiologist, surgeon, and all team (nurses and physiotherapist) to ensure right following protocol and improve treatment. A committed healthcare team is crucial to ensure goal-directed recovery for each patient and achieve specific goals to enhance recovery after oesophagectomy (26). This study suggested that the main condition for adequate implementation of the ERAS protocol is a multidisciplinary team with very experienced and dedicated staff.

This study may be significant because reflects everyday clinical practice and real life. Moreover, the ERAS protocol is feasible even in high-risk patients (with ASA score ≥ 3).

There are a few limitations. The participants were not randomized. Furthermore, there were differences in ASA score and BMI, type of surgical approach, and surgical procedure time between the groups. However, the ASA score was higher in the ERAS group. Despite the difference in BMI, there was no statistically significant difference in NRS-2002 between groups. Modifications in operative technique and approach may impact on clinical outcomes (33, 34), although it has been shown that perioperative protocol with the emphasis on close collaboration between anesthesiologists and other members of a team is an independent factor for successful postoperative outcomes (26).

## 5 Conclusion

The present study showed that implementation of ERAS protocol in an experienced center for esophageal surgery with anesthesiologist elements such as GDT, opioid-sparing anesthesia, and postoperative “near-zero” fluid balance may reduce postoperative morbidity, especially pulmonary complications rate. An implementation of the ERAS protocol after esophagectomy is feasible even in high-risk patients. Evaluation of ERAS protocol through clinical practice and research can obtain more data about the advantages and drawbacks of this approach with special emphasis on fluid management.

## Data availability statement

The raw data supporting the conclusions of this article will be made available by the authors, without undue reservation.

## Ethics statement

The studies involving humans were approved by Ethics Committee of University Clinical Centre of Serbia. The studies were conducted in accordance with the local legislation and institutional requirements. The participants provided their written informed consent to participate in this study.

## Author contributions

MD: Conceptualization, Data curation, Formal analysis, Investigation, Methodology, Writing – original draft. OS: Conceptualization, Investigation, Writing – original draft. DS: Conceptualization, Investigation, Methodology, Writing – review & editing. NK: Writing – review & editing. BM: Data curation, Formal analysis, Methodology, Software, Writing – review & editing. PS: Writing – review & editing. AS: Writing – review & editing. MM: Data curation, Writing – review & editing. SS: Data curation, Writing – review & editing. DM: Data curation, Formal analysis, Writing – review & editing. IP: Conceptualization, Formal analysis, Investigation, Methodology, Supervision, Validation, Writing – original draft.

## References

[1] Bray F Ferlay J Soerjomataram I Siegel RL Torre LA Jemal A. Global cancer statistics 2018: GLOBOCAN estimates of incidence and mortality worldwide for 36 cancers in 185 countries. CA Cancer J Clin. (2018) 68:394–424. doi: 10.3322/caac.21492, PMID: 30207593

[2] Raymond DP Seder CW Wright CD Magee MJ Kosinski AS Cassivi SD. Predictors of major morbidity or mortality after resection for esophageal cancer: a society of Thoracic Surgeons general thoracic surgery database risk adjustment model. Ann Thorac Surg. (2016) 102:207–14. doi: 10.1016/j.athoracsur.2016.04.055, PMID: 27240449 PMC5016796

[3] Tang Z Lu M Qu C Zhang Y Li L Li S. Enhanced recovery after surgery improves short-term outcomes in patients undergoing esophagectomy. Ann Thorac Surg. (2022) 114:1197–11204. doi: 10.1016/j.athoracsur.2021.08.073, PMID: 34624264

[4] Low DE Allum W De Manzoni G Ferri L Immanuel A Kuppusamy M. Guidelines for perioperative care in esophagectomy: enhanced recovery after surgery ERAS. World J Surg. (2019) 43:299–330. doi: 10.1007/s00268-018-4786-4, PMID: 30276441

[5] Scott MJ Baldini G Fearon KC Feldheiser A Feldman LS Gan TJ. Enhanced recovery after surgery (ERAS) for gastrointestinal surgery, part 1: pathophysiological considerations. Acta Anaesthesiol Scand. (2015) 59:1212–31. doi: 10.1111/aas.12601, PMID: 26346577 PMC5049676

[6] Durkin C Schisler T Lohser J. Current trends in anesthesia for esophagectomy. Curr Opin Anaesthesiol. (2017) 30:30–5. doi: 10.1097/ACO.0000000000000409, PMID: 27764049

[7] Nevo Y Arjah S Katz A Ramírez García Luna JL Spicer J Cools-Lartigue J. ERAS 2.0: continued refinement of an established enhanced recovery protocol for esophagectomy. Ann Surg Oncol. (2021) 28:4850–8. doi: 10.1245/s10434-021-09854-7 33774774

[8] White PF Kehlet H Neal JM Schricker T Carr DB Carli F. The role of the anesthesiologist in fast-track surgery: from multimodal analgesia to perioperative medical care. AnesthAnalg. (2007) 104:1380–96. doi: 10.1213/01.ane.0000263034.96885.e1, PMID: 17513630

[9] Taniguchi H Sasaki T Fujita H Kobayashi H Kawasaki R Ogata T. Effects of goal-directed fluid therapy on enhanced postoperative recovery: an interventional comparative observational study with a historical control group on oesophagectomy combined with ERAS program. Clin Nutr ESPEN. (2018) 23:184–93. doi: 10.1016/j.clnesp.2017.10.002, PMID: 29460796

[10] Mukai A Suehiro K Watanabe R Juri T Hayashi Y Tanaka K. Impact of intraoperative goal-directed fluid therapy on major morbidity and mortality after transthoracic oesophagectomy: a multicentre, randomised controlled trial. Br J Anaesth. (2020) 125:953–61. doi: 10.1016/j.bja.2020.08.060, PMID: 33092805

[11] Xing X Gao Y Wang H Qu S Huang C Zhang H. Correlation of fluid balance and postoperative pulmonary complications in patients after esophagectomy for cancer. J Thorac Dis. (2015) 7:1986–93. doi: 10.3978/j.issn.2072-1439.2015.11.24, PMID: 26716037 PMC4669283

[12] Low DE Alderson D Cecconello I Chang AC Darling GE D’Journo XB. International consensus on standardization of data collection for complications associated with esophagectomy: esophagectomy complications consensus group (ECCG). Ann Surg. (2015) 262:286–94. doi: 10.1097/SLA.0000000000001098, PMID: 25607756

[13] Casado D López F Martí R. Perioperative fluid management and major respiratory complications in patients undergoing esophagectomy. Dis Esophagus. (2010) 23:523–8. doi: 10.1111/j.1442-2050.2010.01057.x, PMID: 20459444

[14] Veelo DP van Berge Henegouwen MI Ouwehand KS Geerts BF Anderegg MC van Dieren S. Effect of goal-directed therapy on outcome after esophageal surgery: a quality improvement study. PLoS One. (2017) 12:e0172806. doi: 10.1371/journal.pone.0172806, PMID: 28253353 PMC5333843

[15] Bahlmann H Halldestam I Nilsson L. Goal-directed therapy during transthoracic oesophageal resection does not improve outcome: randomised controlled trial. Eur J Anaesthesiol. (2019) 36:153–61. doi: 10.1097/EJA.0000000000000908 30431499

[16] Rollins KE Lobo DN. Intraoperative goal-directed fluid therapy in elective major abdominal surgery: a Meta-analysis of randomized controlled trials. Ann Surg. (2016) 263:465–76. doi: 10.1097/SLA.0000000000001366 26445470 PMC4741406

[17] Myles PS Bellomo R Corcoran T Forbes A Peyton P Story D. Restrictive versus liberal fluid therapy for major abdominal surgery. N Engl J Med. (2018) 378:2263–74. doi: 10.1056/NEJMoa1801601, PMID: 29742967

[18] Theodorou D Drimousis PG Larentzakis A Papalois A Toutouzas KG Katsaragakis S. The effects of vasopressors on perfusion of gastric graft after esophagectomy. An experimental study. J Gastrointest Surg. (2008) 12:1497–501. doi: 10.1007/s11605-008-0575-y 18612706

[19] Walsh KJ Zhang H Tan KS Pedoto A Desiderio DP Fischer GW. Use of vasopressors during esophagectomy is not associated with increased risk of anastomotic leak. Dis Esophagus. (2021) 34:doaa090. doi: 10.1093/dote/doaa090, PMID: 32944749 PMC8024447

[20] Glatz T Kulemann B Marjanovic G Bregenzer S Makowiec F Hoeppner J. Postoperative fluid overload is a risk factor for adverse surgical outcome in patients undergoing esophagectomy for esophageal cancer: a retrospective study in 335 patients. BMC Surg. (2017) 17:6. doi: 10.1186/s12893-016-0203-9, PMID: 28086855 PMC5237209

[21] Kubo Y Tanaka K Yamasaki M Yamashita K Makino T Saito T. The impact of perioperative fluid balance on postoperative complications after esophagectomy for esophageal cancer. J Clin Med. (2022) 11:3219. doi: 10.3390/jcm11113219, PMID: 35683605 PMC9181193

[22] Chappell D Jacob M Hofmann-Kiefer K Conzen P Rehm M. A rational approach to perioperative fluid management. Anesthesiology. (2008) 109:723–40. doi: 10.1097/ALN.0b013e3181863117, PMID: 18813052

[23] Pillinger NL Kam P. Endothelial glycocalyx: basic science and clinical implications. Anaesth Intensive Care. (2017) 45:295–307. doi: 10.1177/0310057X1704500305, PMID: 28486888

[24] Li X Zhang Q Zhu Y Yang Y Xu W Zhao Y. Effect of perioperative goal-directed fluid therapy on postoperative complications after thoracic surgery with one-lung ventilation: a systematic review and meta-analysis. World J Surg Oncol. (2023) 21:297. doi: 10.1186/s12957-023-03169-5, PMID: 37723513 PMC10506328

[25] Raftery NB Murphy CF Donohoe CL O'Connell B King S Ravi N. The complexity of defining postoperative pneumonia after esophageal Cancer surgery: a Spectrum of lung injury rather than a simple infective complication? Ann Surg. (2022) 276:e400–6. doi: 10.1097/SLA.0000000000004546, PMID: 33201133

[26] Preston SR Markar SR Baker CR Soon Y Singh S Low DE. Impact of a multidisciplinary standardized clinical pathway on perioperative outcomes in patients with oesophageal cancer. Br J Surg. (2013) 100:105–12. doi: 10.1002/bjs.8974, PMID: 23161343

[27] Shanthanna H Ladha KS Kehlet H Joshi GP. Perioperative opioid administration. Anesthesiology. (2021) 134:645–59. doi: 10.1097/ALN.0000000000003572 32991672

[28] Chandrashekar MV Irving M Wayman J Raimes SA Linsley A. Immediate extubation and epidural analgesia allow safe management in a high-dependency unit after two-stage oesophagectomy. Results of eight years of experience in a specialized upper gastrointestinal unit in a district general hospital. Br J Anaesth. (2003) 90:474–9. doi: 10.1093/bja/aeg091, PMID: 12644420

[29] Makaryus R Miller TE Gan TJ. Current concepts of fluid management in enhanced recovery pathways. Br J Anaesth. (2018) 120:376–83. doi: 10.1016/j.bja.2017.10.011 29406186

[30] Huang ZD Gu HY Zhu J Luo J Shen XF Deng QF. The application of enhanced recovery after surgery for upper gastrointestinal surgery: meta-analysis. BMC Surg. (2020) 20:3. doi: 10.1186/s12893-019-0669-3, PMID: 31900149 PMC6942370

[31] Gu CY Zhang J Qian YN Tang QF. Effects of epidural anesthesia and postoperative epidural analgesia on immune function in esophageal carcinoma patients undergoing thoracic surgery. Mol Clin Oncol. (2015) 3:190–6. doi: 10.3892/mco.2014.405, PMID: 25469293 PMC4251267

[32] Beilin B Shavit Y Trabekin E Mordashev B Mayburd E Zeidel A. The effects of postoperative pain management on immune response to surgery. Anesth Analg. (2003) 97:822–7. doi: 10.1213/01.ANE.0000078586.82810.3B 12933409

[33] Bras Harriott C Angeramo CA Casas MA Schlottmann F. Open versus hybrid versus totally minimally invasive Ivor Lewis esophagectomy: systematic review and meta-analysis. J Thorac Cardiovasc Surg. (2022) 164:e233–54. doi: 10.1016/j.jtcvs.2021.12.051, PMID: 35164948

[34] Mariette C Markar SR Dabakuyo-Yonli TS Meunier B Pezet D Collet D. Hybrid minimally invasive esophagectomy for esophageal Cancer. N Engl J Med. (2019) 380:152–62. doi: 10.1056/NEJMoa1805101 30625052

